# The Significance of Enzymatic Cholestasis in Inflammatory Bowel Disease Patients for the Diagnosis of Primary Sclerosing Cholangitis—A Retrospective Study

**DOI:** 10.3390/jcm14165915

**Published:** 2025-08-21

**Authors:** Matei Mandea, Dragos M. Oancea, Mihaela C. Ghioca, Speranta M. Iacob, Razvan A. Iacob, Ioana. G. Lupescu, Liliana S. Gheorghe

**Affiliations:** 1 Department of Internal Medicine, Discipline of Gastroenterology and Hepatology, University of Medicine and Pharmacy Carol Davila, 020021 Bucharest, Romania; dragos-mihai.oancea@drd.umfcd.ro (D.M.O.); drlgheorghe@gmail.com (L.S.G.); 2 Digestive Diseases and Liver Transplant Center, Fundeni Clinical Institute, 022328 Bucharest, Romania; mihaela.lita@drd.umfcd.ro; 3 Department of Radiology, Medical Imaging and Interventional Radiology I, University of Medicine and Pharmacy “Carol Davila”, 020021 Bucharest, Romania

**Keywords:** primary sclerosing cholangitis, retrospective study, inflammatory bowel disease, enzymatic cholestasis, gamma-glutamyl transpeptidase, liver diseases

## Abstract

This retrospective longitudinal study evaluated the significance of cholestasis syndrome and the diagnosis of primary sclerosing cholangitis (PSC) in inflammatory bowel disease (IBD) patients from a tertiary center in Romania. **Methods:** From 2011 to 2022, 3767 patients suspected for IBD were evaluated, with 2499 confirmed cases. Of these, 34 patients (1.36%) had an IBD-PSC phenotype. Of the IBD-PSC cases, 56% were associated with UC and 44% with CD. **Results:** Enzymatic cholestasis was observed in 13.3% of IBD patients, with gamma-glutamyl transpeptidase (GGT) elevated in 70.2% and alkaline phosphatase (ALP) in 51.3%. However, only 10.2% of the patients with enzymatic cholestasis were diagnosed with PSC. Other liver diseases identified included metabolic-associated steatotic liver disease (MASLD), chronic viral hepatitis, Primary Biliary Cholangitis, autoimmune hepatitis, and liver neoplasms. A higher incidence of cholangiocarcinoma (11.76% vs. 0.24%, *p* < 0.001) and liver-related death (8.82% vs. 0.65%, *p* < 0.001) was found between IBD-PSC patients and those without PSC. PSC-CD patients were diagnosed at a younger age (30.2 vs. 43 years, *p* < 0.001), had higher rates of severe disease (73.3% vs. 10.5%, *p* < 0.001), required more biological treatment (60% vs. 15.7%, *p* < 0.001), and experienced higher mortality (20% vs. 0%, *p* < 0.001). **Discussions:** This study represents the most extensive cohort analysis of PSC-IBD patients in Romania and Eastern Europe, highlighting clinical differences between PSC-UC and PSC-CD phenotypes. **Conclusions:** The regular monitoring of ALP and GGT in IBD patients helps detect liver diseases, including PSC. However, only one in ten patients with IBD and enzymatic cholestasis was diagnosed with PSC.

## 1. Introduction

The association of primary sclerosing cholangitis (PSC) and inflammatory bowel diseases (IBD) is often described as a distinctive entity representing a special phenotype. While PSC affects the intra- and extrahepatic bile ducts, which appear irregular, with multifocal strictures, IBDs can affect any segment of the intestinal tract. Still, the most commonly affected part is the colon, especially in the setting of coexisting PSC. Moreover, both diseases share some common characteristics: the presence of chronic inflammation, an autoimmune pathogenesis, and an increased risk of malignancy (cholangiocarcinoma—CCA or colorectal cancer—CRC) [1,2,3,4].

PSC is characterized as a chronic cholestatic disease, with the inflammation and progressive fibrosis of the bile ducts ultimately leading to liver cirrhosis and liver failure [5,6].

IBDs represent a group of immune-mediated chronic conditions characterized by inflammation of the gastrointestinal system. The IBD most frequently diagnosed together with PSC is ulcerative colitis (UC), seen in 80–90% of patients [7,8]. The overall prevalence of IBD in PSC is 60–80% of the patients [8,9]. The epidemiology and association of IBD with PSC are well known at the European level, especially in Western European and Nordic countries. A comparison of recent cohort studies from different parts of the world showed a stronger link between PSC and IBD in populations of North America, Australia, and Northern Europe (60–78%), while the association was weaker in Southern Europe and Southeast Asia (20–53%). The prevalence of PSC in IBD patients is variable, as Barberio et al. reported in a meta-analysis, as being 2.16% globally. Other studies reported values ranging from 0.12% to 8.1%, with lower values in Eastern Asia (average of 0.6%) and Southern Europe (average of 0.99%) and higher values in North America (3.58%) and Finland (10.97%) [3,4,9,10,11,12]. Regarding patient progression and PSC severity, it was more severe in patients with IBD-PSC in Northern Europe, Asia, and North America, with a younger age at diagnosis, and was similar between groups in the Southern European population, but with longer survival in European patients compared to those from Asia and North America [3,9,10,13]. Concerning the extent and severity of IBD, UC-PSC with mild pancolitis was more common in North America, Northern Europe, Asia, and Australia, and less common in Southern Europe [9,12,14].

The importance of screening for PSC is to improve the prognosis by adapting therapy to each type of patient [15,16].

Several common pathogenic mechanisms have been proposed for the two pathologies, such as genetic risk, immune-mediated pathways, and gut microbiome changes, but none have yet been confirmed. Furthermore, changes in the liver–gut axis induced by environmental factors can lead to pathologic conditions in both the liver and the gut [17,18,19].

Enzymatic cholestasis in patients with IBD can be assessed by measuring the serum level of bile salts, bilirubin, alkaline phosphatase (ALP), and/or gamma-glutamyl transpeptidase (GGT). Other than PSC, cholestasis can have multiple causes, such as drug-induced causes and other autoimmune liver diseases like Primary Biliary Cholangitis (PBC), Autoimmune Hepatitis (AIH), or persistent inflammation [20,21].

## 2. Materials and Methods

This retrospective study included 3767 patients evaluated for IBD in our clinic (Digestive Diseases and Hepatic Transplant Center, Fundeni Clinical Institute, Bucharest) between 2011 and 2022. Patients were added to the hospital’s electronic management system; evaluated using clinical, biochemical, and paraclinical standard tests; and diagnosed with a form of IBD and/or PSC. Data from PSC patients diagnosed in the same period, regardless of the association with IBD, was then analyzed. This data included clinical, laboratory, radiologic, and endoscopic information for diagnosis and follow-up.

The evaluation of patients suspected of having IBD was performed according to European protocols, with respect to endoscopic, histopathological, and imaging evaluations. In addition, the hospital protocol requires evaluation for the presence of cholestasis every 6 months.

The diagnosis of PSC was made in the setting of chronic cholestasis (elevated gamma-glutamyl transpeptidase and/or alkaline phosphatase), typical Magnetic Resonance Cholangiopancreatography (MRCP) findings, or typical histological findings for small-duct PSC in the absence of specific biomarkers for other pathologies.

The diagnosis of PSC was established by performing an MRCP examination, which revealed strictures and dilatations of the biliary tree, with exclusion of causes of Secondary Sclerosing Cholangitis. Patients who presented a normal MRCP exam underwent liver biopsy to establish the diagnosis.

The purpose of this study was to identify how sensitive the presence of cholestasis syndrome is in predicting PSC in patients with IBD. The secondary objectives were to assess the progression and severity of disease in patients with IBD-PSC phenotype and in those without PSC, as well as to evaluate what other chronic liver conditions affect patients with IBD.

The severity of IBD was evaluated using clinical criteria, such as whether the patient had surgery, biologic therapy, multiple courses of corticosteroids, or extended hospitalization. Any of these factors documented in the patient’s medical records were considered an indicator of severe disease.

Data analysis was performed using DataTAB (Online Statistics Calculator, DataTab e.U. Graz, Austria; URL https://datatab.net/) and SPSS (version 26.0; Chicago, IL, USA). Joint data were presented using mean and interquartile ranges and assessed using the Mann–Whitney U test or independent samples *t*-test, as appropriate. Differences in proportions were analyzed using the Chi-square test or the exact Fisher test, as appropriate.

## 3. Results

**Figure 1:**
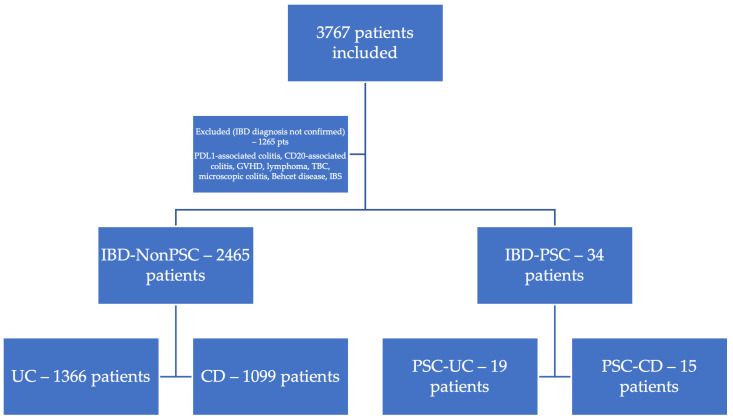
Inclusion of patients with Inflammatory Bowel Disease.

From 2011 to 2022, 3767 patients suspected of IBD were subsequently evaluated. As shown in Figure 1, 2499 were confirmed with IBD (66.33%). Following the evaluation, 1265 patients were excluded, as they were instead diagnosed with Irritable Bowel Syndrome (IBS), anti-PDL 1 or anti-CD20 antibody immunotherapy-associated colitis, graft-versus-host disease (GVHD), intestinal lymphoma, Burkitt lymphoma, intestinal tuberculosis, microscopic colitis, or Behcet’s disease. Among the patients with confirmed IBD, 2465 (98.52%) had no associated PSC, and 34 patients presented an IBD-PSC phenotype. Among the patients with IBD without PSC, 1366 (55.42%) were diagnosed with UC and 1099 with CD (44.58%). Among the patients with IBD-PSC, 15 (44%) were diagnosed with CD and 19 with UC (56%).

**Figure 2:**
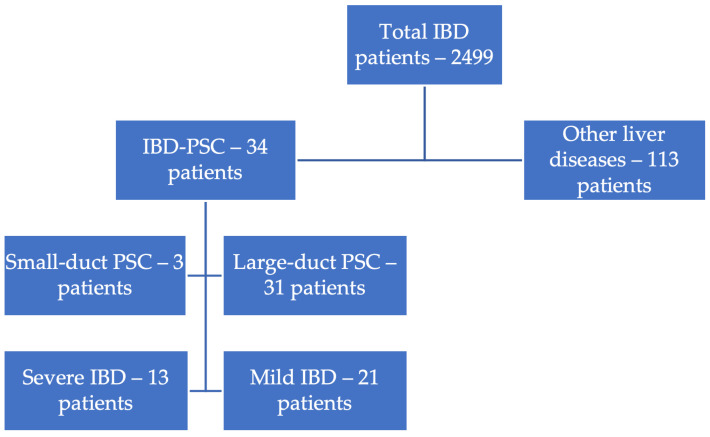
IBD severity, phenotype, and liver involvement.

Figure 2 shows all patients with IBD and identified liver damage. Thus, out of 2499 patients with IBD, 147 (5.88%) had a diagnosis of liver disease. Of these, 34 (23.2%) were diagnosed with PSC and 113 (76.8%) with other liver diseases. It is important to mention that 333 (13.3%) patients with IBD had enzymatic cholestasis syndrome (ECS, defined as GGT above ULN = 55 U/L or ALP above ULN = 120 U/L), with GGT being increased in 70.2% of these patients and ALP increased in 51.3% of these patients.

It is worth mentioning that among the patients with IBD and coexisting liver disease, 1.2% had metabolic-associated steatotic liver disease (MASLD), 2.67% had chronic viral hepatitis (B or C viral hepatitis), three patients had PBC, four had AIH, and 0.51% had a liver neoplasm (hepatocarcinoma or CCA).

Table 1 shows the comparison between all patients with IBD without PSC and those with IBD and PSC. The second category accounted for 1.36% of the total patients. A lower age at diagnosis was observed for patients with IBD-PSC, 37.35 years, compared to those without PSC; nonetheless, the difference was not statistically significant (*p* = 0.059). In terms of the incidence of CCA or mortality due to liver disease, significantly more cases have been observed among patients with the IBD-PSC phenotype (*p* < 0.001). Patients with ECS had a mean GGT value of 275.53 U/L (95%CI 42.4–508.6) and a mean ALP value of 347.4 U/L (95%CI 211.6–483.1), which is significantly higher in patients with PSC (*p* < 0.001). Moreover, using the logistic regression analysis, the probability of a PSC diagnosis significantly increased when ALP values exceeded 332 U/L, OR = 1.018 (95%CI 1.014–1.022). The ROC Curve Analysis showed an area under the ROC curve (AUROC) of 0.937, with a sensitivity of 99.7% and specificity of 32.3% for the cut-off.

Table 2 shows that there were significant differences between patients with PSC-UC and those with UC without PSC, with a higher incidence of CCA, a higher need for biologic therapy, a higher rate of severe IBD, and higher mean values for cholestasis markers in those with PSC-UC.

It should be noted that in total, 13.3% (333) of the patients diagnosed with IBD had enzymatic cholestasis, of which 19.8% had an underlying liver disease, with 78.37% (261) of them undergoing the MRCP examination. A total of 21.62% (72) of the patients with ECS did not undergo further investigations for PSC (neither MRCP nor liver biopsy)

Table 3 showcases demographic and clinical data comparing patients with Crohn’s Disease (CD) and PSC (PSC-CD) to those without PSC (CD non-PSC). The mean age is significantly lower (*p* < 0.05) in PSC-CD patients (30.2 years) compared to CD non-PSC patients (36.08 years), indicating that PSC may manifest at a younger age in CD patients. The distribution of patients with CD using the Montreal Classification shows no significant differences between the groups in terms of the disease extension and behavior. Severe disease occurrence and the use of biological treatments are somewhat more common in PSC-CD patients, though not statistically significant. Markers of enzymatic cholestasis (GGT and ALP levels) are significantly higher (*p* < 0.001) in the PSC-CD group, underlining the liver’s involvement in these patients.

Briefly comparing the two groups of patients, PSC-UC and PSC-CD, we can see in Table 4 that the mean age at diagnosis for IBD is significantly higher in PSC-UC patients. PSC-UC patients have a lower rate of severe IBD, the need for biological treatment, and death compared to PSC-CD patients, indicating potential differences in disease progression and severity between UC and CD when associated with PSC.

We also conducted a separate analysis of patients diagnosed with PSC within the same time frame (2011–2022), comprising 100 patients. Among these, 60.56% were female (*p* = 0.063). Notably, the age at diagnosis of non-IBD PSC patients was significantly higher, with a mean of 45.37 (SD 15.38; *p* = 0.02). A liver transplantation was performed in 19.72% of patients (*p* = 0.257), while liver-related death occurred in 18.31% of patients (*p* = 0.324). The mean enzymatic cholestasis at diagnosis was 306.74 U/L for ALP (95%CI 222.96–390.53, *p* = 0.867) and 291.8 U/L for GGT (95%CI 204.37–379.23, *p* = 0.334).

## 4. Discussion

The main objective of this study was to evaluate the evolution and characteristics of liver disease in PSC patients with or without IBD, consistent with the experience in our center.

It is important to mention that this study assembles one of the largest Eastern European series of PSC-IBD patients to date, as it is the first cohort study performed on a larger population from a large tertiary care center for liver diseases and inflammatory bowel diseases from Romania, which evaluated PSC in IBD patients over 11 years.

The results presented show that not all patients with IBD and an increase in liver cholestasis enzymes had underlying liver damage, and only 1/10 of these were diagnosed with PSC. Given that this longitudinal study was conducted on a population already diagnosed with IBD, there are no data on patients who had PSC before the diagnosis of IBD.

Previously published data on the incidence of PSC in patients with IBD have shown that it is more common in patients with UC, those with the PSC-UC phenotype being much more common than those with PSC-CD, as shown by a meta-analysis performed by Barberio et al., which found a 1.7 times higher incidence of PSC in IBD patients with UC [8,10,13]. These data are similar to those observed in our study, but with a higher proportion of patients with PSC-CD, as the incidence of PSC-UC was 1.26 times greater. This higher incidence of CD-PSC is similar to other studies in Southern Europe [3,9]. It was not caused by a referral bias, as this is the most important IBD center in Romania. Among the cohort, there were fewer patients with CD than with UC. We acknowledge that the actual prevalence of PSC in IBD might be underestimated, as some patients were diagnosed based on the MRCP exam despite not showing ECS in hospital tests, and there is a possibility that some of the patients with ECS were not diagnosed with PSC, considering that only 78.37% of these patients underwent MRCP. This aspect is critical as it might influence not only the diagnosis of PSC but also the evolution of patients [22,23].

When analyzing the type of PSC, we noticed that most of the patients had large-duct PSC, with the proportion of small-duct PSC being comparable to the literature, between 5% and 20% of patients with PSC [6,24].

Following the analysis of data on IBD-PSC patients and non-PSC IBD patients, not only were there statistically significant differences in the degree of enzymatic cholestasis and the incidence of CCA (both higher in the PSC group), but also in disease-related death, including from IBD and liver injury. This result is not surprising, given that the risk of CCA was previously described as significantly higher in this population, and an annual MRCP follow-up is still recommended by the latest EASL guideline [5,23,25]. Moreover, the age at diagnosis for gastrointestinal autoimmune disease was marginally lower in the IBD-PSC population. An interesting aspect of the IBD-PSC population was the higher percentage of females in this population. These data differ from most studies, which report that patients are mainly male, except for the study by Kuo et al., which found a percentage similar to our data, at 52% [14,26,27].

Regarding the assessment of ECS, ALP, and GGT were used. However, the latter is not a common marker used in studies for the diagnosis of enzymatic cholestasis, as it is less sensitive for cholestatic diseases and may be influenced by other conditions, the most important of which is a metabolic insult to the liver. However, there are studies and also expert opinions that show that it should not be neglected when evaluating a patient with a possible cholestatic liver injury [25,27,28].

The analysis of the comparative data between the two groups of PSC-UC and PSC-CD, compared to the non-PSC group, showed slight differences compared to the data presented by Barberio et al., who found a predominantly pancolonic involvement in the PSC-UC group, compared to our population, where most of the patients had left colonic involvement. At the same time, in the PSC-CD subgroup of patients, the extent of the intestinal disease was similar to previously reported data, with disease limited to the upper digestive tract being rare [4,8,13,26].

Although previous data showed that patients with the PSC-CD phenotype had a milder form of the disease, with a lower rate of CCA, our results showed the opposite, with a lower age at diagnosis, a more severe form of IBD, a higher need for biological treatment, and a higher rate of mortality for those with PSC-CD compared to those with PSC-UC. The clinician should increase their focus on ECS in PSC-CD patients, and diagnostic tests such as blood markers, MRCP, or liver biopsies should be performed promptly [2,10,13,29].

Hepato-biliary manifestations in patients with inflammatory bowel disease can be common, and abnormal ALP and GGT levels can be benign. However, they can also indicate an underlying liver injury and lead to a diagnosis of chronic liver disease, as observed in 5.88% of the IBD patients, with our data being comparable to the data presented by Gaspar et al. [30]. Both ALP and GGT lack specificity for diagnosing liver diseases, including cholestatic conditions, but may indicate liver disorders. They are indicative of PSC in about 75% of patients, as PSC can be detected in IBD patients via MRCP, even without biochemical cholestasis [22,25]. ALP can rise in children during growth and in autoimmune liver diseases like PBC, AIH, IgG4 sclerosing cholangitis, as well as in cirrhosis, cholangiocarcinoma, hepatocarcinoma, and acute cholangitis or lithiasis [1,22,23,31,32]. GGT levels may be elevated in biliary lesions in children, liver cirrhosis, toxic liver injury, PBC, AIH, IgG4 sclerosing cholangitis, or MASLD [23,33,34]. Our analysis showed IBD patients may have other autoimmune liver diseases like PBC or AIH [22,25]. 

Compared to other cohort studies, the prevalence of PSC in IBD patients was less than 2.16%, as Barberio et al. reported globally, and more comparable with Southern Europe [3,4,9,10,11,12]. The PSC severity was higher in IBD-PSC patients from Northern Europe, Asia, and North America, who were diagnosed at a younger age, and it was similar in Southern Europe. However, survival was longer in Europe compared to Asia and North America. UC-PSC with mild pancolitis was more common in North America, Northern Europe, Asia, and Australia, and less common in Southern Europe [3,9,10,13,14]. Therefore, our cohort data is similar to that of the previously described Southern European population, showing a lower link to IBD, a younger age at diagnosis in PSC-IBD, and a weaker association with UC-PSC, which does not involve the entire colon [3,9].

Our study had limitations. Because our experience with PSC patients is evolving, consistent with the increasing incidence in recent years, we lack a standard of management and follow-up for these individuals, such as standard periodic MRCP for every IBD patient with enzymatic cholestasis, leading to the possibility of misreporting complications. Even if we collected data from the hospital database regardless of the evolution and complications, we face the inherent bias of an observational retrospective report [8,14,35].

There is a strong link between PSC and IBD. Although the incidence is variable, this association remains important, even though the underlying mechanisms behind the occurrence of both pathologies remain unknown. A special pattern is seen in these patients, with a milder IBD course, a predominantly colonic involvement, and a higher risk of developing complications [4,23]. In the future, screening and follow-up should be more focused on the use of international guidelines for the standard of care and evaluation.

## 5. Conclusions

This retrospective longitudinal study conducted on a large cohort of patients evaluated for IBD in a large center specialized in the diagnosis and treatment of patients with IBD showed that periodic evaluations by the monitoring of cholestasis enzymes may be useful, leading to an increase in the rate of liver disease diagnoses, which is a special consideration in this population, given the inherent predisposition and vulnerability to liver afflictions.

Our work showed that in the Romanian population, there is a lower risk of a PSC diagnosis in IBD patients. However, when PSC is associated with CD, it is more severe, diagnosed at a younger age, and more often associated with liver-related death.

This study shows that a significant degree of enzymatic cholestasis is needed for the diagnosis of PSC. Only one in ten patients with IBD and enzymatic cholestasis was diagnosed with PSC.

## Figures and Tables

**Table 1:** Comparison between IBD-PSC patients and IBD non-PSC patients.

Value	Inflammatory Bowel Disease Patients		
	IBD-PSC	IBD Non-PSC	*p* Value
Mean age, years (95%CI)	37.35 (32.4–42.2)	42.87 (42.2–43.5)	0.059
Female sex %	52.94%	46.43%	0.450
Severe IBD %	35.25%	34.73%	0.710
Cholangiocarcinoma %	11.76%	0.24%	<0.001
Colonic surgery %	11.76%	14.59%	0.643
Mean ALP U/L	308.82 (232.4–385.1)	79.21 (77.5–80.8)	<0.001
Mean GGT U/L (95%CI)	243 (134.9–351.1)	30.44 (28.5–32.3)	<0.001
Disease-related death %	8.82%	0.65%	<0.001

**Table 2:** Demographic and clinical data comparison between PSC-UC patients and UC non-PSC patients; * GGT ULN = 55 U/L; ** ALP ULN = 120 U/L.

	Ulcerative Colitis Patients (*n* = 1386)		
	PSC-UC (*n* = 19)	UC Non-PSC (*n* = 1367)	*p* Value
Mean age, years (95%CI)	43 (36.1–49.8)	48.3 (47.4–49.2)	<0.001
Female sex %	45.72%	47.37%	0.880
Montreal E1 %	26.12%	5.26%	0.039
Montreal E2 %	51.94%	15.79%	0.002
Montreal E3 %	21.87%	78.95%	<0.001
Severe disease %	23.34%	10.53%	0.189
Biological treatment %	22.75%	15.79%	0.472
Azathioprine treatment %	26.12%	31.58%	0.591
5-ASA treatment %	95.61%	100%	0.350
Liver disease %	-	4.17%	-
CCA %	10.5%	0.43%	<0.001
CRC %	15.79%	0.88%	<0.001
Colonic surgery	5.26%	5.56%	0.955
Mean GGT, U/L (SD) *	217.32 (190.7)	31.76 (54)	<0.001
Mean ALP, U/L (SD) **	278.37 (196.8)	78.12 (41.71)	<0.001

**Table 3:** Demographic and clinical data comparison between PSC-CD patients and CD non-PSC patients; * GGT ULN = 55 U/L; ** ALP ULN = 120 U/L.

	Crohn’s Disease Patients (*n* = 1116)		
	PSC-CD (*n* = 15)	CD Non-PSC (*n* = 1101)	*p* Value
Mean age, years (95%CI)	30.2 (24.3–36.1)	36.08 (35.1–36.9)	0.043
Female sex	60%	47.32%	0.329
Montreal Classification			
Extension			
L1	13.3%	21.8%	0.429
L2	46.67%	43.87%	0.828
L3	40%	33.79%	0.614
L4	0.27%	0.27%	0.840
Behavior			
B1	60%	59.95%	0.997
B2	33.33%	31.34%	0.868
B3	20%	12.99%	0.424
Perianal disease	13.33%	16.35%	0.754
Severe disease	73.33%	49.95%	0.671
Biological treatment	60%	54.5%	0.072
Azathioprine treatment	66.67%	48.96%	0.173
5-ASA treatment	73.33%	59.4%	0.275
Liver disease	-	5.02%	-
CCA	2	1	-
CRC	0	1.25%	-
Enterocolonic surgery	20%	25.45%	0.610
Mean GGT, U/L (95%CI) *	275.53 (42.4–508.6)	28.87 (26.9–30.7)	<0.001
Mean ALP, U/L (95%CI) **	347.4 (211.6–483.1)	80.51 (78.4–82.5)	<0.001

**Table 4:** Main comparison between PSC-UC and PSC-CD patients. * GGT ULN = 55 U/L; ** ALP ULN = 120 U/L.

	PSC-UC (*n* = 19)	PSC-CD (*n* = 15)	*p* Value
Mean age	43	30.2	0.005
CCA	10.5%	13.3%	0.801
Biological treatment	15.7%	60%	0.007
Death	0	20%	0.041
Severe IBD	10.5%	73.3%	<0.001
Mean GGT, U/L (95%CI) *	217.3 (125.4–309.2)	275.5 (42.4–508.6)	0.594
Mean ALP, U/L (95%CI) **	278.37 (183.4–373.2)	347.4 (211.6–483.1)	0.369

## Data Availability

The data presented in this study are available on request from the corresponding author due to ethical and legal restrictions.

## Abbreviations

The following abbreviations are used in this manuscript:

PSC	Primary Sclerosing Cholangitis
IBD	Inflammatory Bowel Disease
UC	Ulcerative Colitis
CD	Crohn’s Disease
GGT	Gamma-Glutamyl Transpeptidase
ALP	Alkaline Phosphatase
MASLD	Metabolic-Associated Steatotic Liver Disease
MRCP	Magnetic Resonance Cholangiopancreatography
ECS	Enzymatic Cholestasis Syndrome
ULN	Upper Limit of Normal
IBS	Irritable Bowel Syndrome
PDL 1	Programmed Death-Ligand 1
GVHD	Graft-Versus-Host Disease
PBC	Primary Biliary Cholangitis
CCA	Cholangiocarcinoma
CRC	Colorectal Cancer
5-ASA	5-Aminosalicylic Acid
AIH	Autoimmune Hepatitis
SD	Standard Deviation
EASL	European Association for the Study of the Liver
U/L	Units per Liter
